# Subjective well-being and its influencing factors of clinical nurses: a cross-sectional study based on latent profiles analysis

**DOI:** 10.3389/fpubh.2026.1969381

**Published:** 2026-10-01

**Authors:** Yanjun Zhao, Fangyan Yan, Ping Ma, Haiying Sui, Yunying Lin

**Affiliations:** Weifang University of Science and Technology, Weifang, China

**Keywords:** clinical nurse, influencing factors, latent profiles analysis, psychological reward satisfaction, subjective well-being

## Abstract

**Objective:**

To investigate the latent class characteristics of subjective well-being among clinical nurses, analyze the influencing factors of different classes, and provide a reference for developing targeted intervention measures.

**Methods:**

Using a convenience sampling method, 358 clinical nurses were recruited from five tertiary general hospitals in Weifang, Shandong Province, between September and December 2025. A general information questionnaire, the General Well-Being Schedule, and the Psychological Reward Satisfaction Scale were used for the survey. Latent profile analysis was employed to establish the latent class model of clinical nurses’ subjective well-being, and multivariate logistic regression was used to analyze the influencing factors of different subjective well-being classes.

**Results:**

Clinical nurses’ subjective well-being could be classified into three latent classes: the Low Well-being-Emotional Control Group (11.9%), the Moderate Well-being-Overall Fluctuation Group (61.0%), and the High Well-being-Health Concern Group (27.2%). Work intensity, family economy, department, whether to engage in nursing again, and psychological reward satisfaction were influencing factors of different latent profiles of clinical nurses’ subjective well-being (*p* < 0.05). A 1-point increase in psychological reward satisfaction was associated with a 14.0% decrease in the odds of being classified into the Low Well-being-Emotional Control Group (OR = 0.860, 95% CI: 0.798–0.927), indicating that psychological reward satisfaction served as an independent protective factor.

**Conclusion:**

There is significant heterogeneity in clinical nurses’ subjective well-being. Nursing managers should pay special attention to clinical nurses in the “Low Well-being-Emotional Control Group” and implement interventions based on the influencing factors of different latent profiles to improve their subjective well-being.

## 1 Introduction

Happiness is an eternal topic. It exists in every corner of people’s daily lives and directly affects their quality of life and mental health (1). Ryan et al. (2) argue that there are two basic views of happiness in psychology. One view defines happiness as pleasure, referring to life satisfaction and the attainment of pleasant experiences and the avoidance of pain, namely the hedonic view. The other view emphasizes life meaning, self-knowledge, and self-actualization, defining happiness as the degree of perfection in personal functioning, namely the eudaimonic view. The definitions of happiness mainly encompass two orientations: subjective well-being and psychological well-being. Wilson (3) was among the first to conduct research on subjective well-being, suggesting that happy individuals should possess traits such as optimism, health, and wisdom. In recent years, subjective well-being has increasingly become a focal point of scholarly research.

Subjective Well-Being (SWB) refers to an individual’s overall perception and evaluation of their quality of life based on their own subjective criteria (4). In the high-stress, high-workload hospital environment, nurses, as the primary point of direct contact with patients and their families, require urgent attention to their subjective well-being (5). Nurses’ well-being not only affects their own health but also influences their work behaviors and performance, which in turn impacts patients. Research has shown (6) that nurses’ SWB is closely associated with the quality of nursing care and patient safety. Enhancing nurses’ SWB levels is of great significance for stabilizing the nursing workforce, improving the quality of care, and enhancing patients’ healthcare experiences (7).

Psychological reward, as a motivational strategy, enables nurses to feel recognized and cared for amidst their busy nursing work, thereby mobilizing their work enthusiasm and stimulating their work potential to a certain extent (8). Psychological reward satisfaction (PReSS) refers to the supportive and positive evaluative outcomes that employees obtain from professional interpersonal relationships established with their clients, colleagues, or supervisors, including trust, recognition, and praise (9). Psychological rewards can come in the form of a caring greeting or a word of praise from a leader. When employees feel exhausted, a leader’s simple words “You have worked hard” can bring them great psychological comfort! When employees successfully complete a task, a leader’s heartfelt “Well done” can give them tremendous encouragement! Psychological reward serves as a necessary supplement to the limited financial compensation available in hospitals (10). Improving nurses’ psychological reward satisfaction is conducive to reducing their turnover intention, enhancing innovative behaviors, and increasing their work engagement and professional identity (11).

Currently, there are relatively few studies on nurses’ SWB in China, and the research subjects have been limited to emergency department, ICU, and outpatient nurses (12–14), with a lack of studies covering the entire nursing workforce. Moreover, in Chinese research on nurses’ subjective well-being, most studies have used the total scale score to determine the level of SWB, neglecting the heterogeneity among individuals. Latent profile analysis (LPA) is a statistical method that classifies individuals into different latent subgroups based on their response patterns on observed variables (15). Therefore, this study employs LPA to classify nurses’ subjective well-being and to explore the characteristics of different classes and their influencing factors. The aim is to help hospital administrators accurately identify nurse groups with low SWB and implement targeted group interventions, thereby comprehensively enhancing nurses’ SWB levels, optimizing the quality of nursing care, and improving patients’ healthcare experiences.

## 2 Objects and methods

### 2.1 Study population

Using a convenience sampling method, clinical nurses from five tertiary hospitals were selected as research subjects from September to December 2025. Inclusion criteria were: ① registered nurses who had obtained a nurse practice qualification certificate; ② nurses who had been engaged in clinical nursing work for ≥1 year; ③ nurses who voluntarily participated in this study and signed informed consent forms. Exclusion criteria were: ① intern nurses and training nurses; ② nurses who were not on duty during the survey period. A total of 19 variables were included in this study. The sample size was calculated as 5–10 times the number of variables, with an additional 20% allowance for invalid questionnaires, resulting in a required total sample size of 114–228 cases. The actual sample size included in this study was 358. This study was reviewed and approved by the Ethics Committee of Weifang University of Science and Technology (approval No. HL2026008) on August 14, 2025.

### 2.2 Survey instruments

#### 2.2.1 The general information questionnaire

The demographic questionnaire was self-designed based on the purpose of the study, including 9 questions about the participants’ sex, age, marital status, education level, work intensity, monthly income, department, family economy and whether to engage in nursing.

#### 2.2.2 General well-being schedule

Subjective well-being was measured by the Generall Well-being Schedule (GWB) developed by Fazio (16), translated and revised by Chinese scholar Duan (17). This scale has been widely used at home and abroad with good reliability and validity. The Cronbach’s α of the scale is 0.816. The scale includes 18 items in total. The scale employed a mixed scoring system: items 2, 5, 6, and 7 used a 5-point Likert scale (1–5); items 15–18 used an 11-point scale (0–10); and the other items used a 6-point scale (1–6). Total scores ranged from 14 to 120, with higher scores reflecting greater subjective well-being. TThe scale consists of 6 dimensions, including 2 items of health worry (items 10 and 15), 4 items of energy level (items 1, 9, 14 and 17), 2 items of satisfying interesting life (items 6 and 11), 3 items of depressed-cheerful mood (items 4, 12 and 18), 3 items of emotional-behavioural (items 3, 7 and 13), and 4 items of relaxed versus tense anxious (items 2, 5, 8 and 16). The Cronbach’s α coefficient for this scale in the present study was 0.834.

#### 2.2.3 Psychological reward satisfaction scale (PReSS)

Psychological reward satisfaction was measured by the Psychological Reward Satisfaction Scale (PReSS) developed by Gieter (18), translated and revised by Chinese scholar Luo (19). The scale has 12 items and 3 dimensions, which are, respectively, used to measure nurses’ psychological reward satisfaction from head nurses, doctors and patients. The three dimensions have good internal consistency. The answers are scored by Likert1-5, which, respectively, represent very dissatisfied, dissatisfied, uncertain, satisfied and very satisfied. The total score of the scale ranged from 12 to 60, with higher scores reflecting greater satisfaction with psychological rewards among the respondents. The Cronbach’s α for this scale in the present study was 0.937.

### 2.3 Data collection

First, the investigators contacted the hospital’s nursing department to describe the purpose and process of the study, and obtained their support. Then, the researchers made the survey content of this study (covering sociodemographic information, subjective well-being andpsychological reward satisfaction) into an electronic questionnaire, and conducted the survey through WeChat, so that every qualified hospital nurse could receive the questionnaire, these nurses choose whether to fill in the questionnaire completely according to their own subjective wishes (an electronic consent form was presented on the questionnaire’s front page, and participants could only start the survey after clicking “I have read and agree to participate”). After the questionnaires are collected, the questionnaires are checked one by one, and the incomplete questionnaires and the questionnaires with obvious rules (questionnaires with identical responses across all items or with a fixed repeated pattern (e.g., 1–2–3-4-5).) are eliminated to ensure the validity of the data. In total, 376 questionnaires were returned. After the removal of 18 invalid questionnaires, 358 valid responses were included in the final analysis, representing a valid response rate of 95.21%.

### 2.4 Statistical analysis

Mplus 8.3 software was used to analyze the latent profiles of clinical nurses’ subjective well-being. Starting from a single-class model, the number of classes was gradually increased, and models were compared based on fit indices of latent profile analysis to select the optimal class model. The commonly used fit indices are as follows: ① Model evaluation indices: Akaike information criterion (AIC), Bayesian information criterion (BIC), sample size-adjusted BIC (aBIC), and Entropy; ② Model comparison indices: Lo–Mendell–Rubin (LMR) and bootstrap likelihood ratio test (BLRT). Among these, AIC, BIC, and aBIC all evaluate model fit by comparing the differences between observed and expected values, with smaller values indicating better model fit. The Entropy index reflects the classification accuracy of the model, ranging from 0 to 1, with values closer to 1 indicating more precise classification. LMR and BLRT are used to compare the fit differences between different latent profile models, and a significant *p*-value for these tests indicates that the k-class model fits better than the k-1-class model (15).

SPSS 26.0 was used for statistical analysis of the data. Continuous variables conforming to normal distribution were expressed as mean ± standard deviation ( $\bar{X}$ ±S), while continuous variables not conforming to normal distribution were expressed as median (interquartile range) [M (P25, P75)]. Categorical variables and ordinal variables were expressed as frequencies and percentages (%). One-way analysis was used to examine the distribution differences of different subjective well-being classes across general demographic data and each scale. A logistic regression model with cluster-robust standard errors was fitted, with the latent profile classification as the dependent variable, and the covariates that showed statistically significant associations in the univariate analyses entered as independent variables. A *p*-value < 0.05 was considered statistically significant.

## 3 Results

### 3.1 Description of participants

Among the 358 participants, 94.7%(339) were females, 71.2%(255) were married, 72.1% obtained a undergraduate degree, 57.8% were over 30 years old, only 10.6% earned less than RMB 4,000 per month, 77.6% reflected that the work intensity is big, 35.5% would not choose nursing jobs again. Table 1 displays the details.

**Table 1:** Univariate analysis among the three classes (*n* = 358).

Variables	*N*	Low Well-being-Emotional control group (*n* = 42)	Moderate Well-being-Overall fluctuation Group (*n* = 218)	High Well-being-Health concern group (*n* = 98)	Inspection system calculates	*P*
Gender					*χ*^2^ = 9.982	0.007
Male	19 (5.3)	1 (2.4)	18 (8.3)	0 (0.0)		
Female	339 (94.7)	41 (97.6)	200 (91.7)	98 (100.0)		
Age (years)					*χ*^2^ = 11.012	0.201
21–25	56 (15.6)	4 (9.5)	39 (17.9)	13 (13.3)		
26–30	95 (26.5)	11 (26.2)	60 (27.5)	24 (24.5)		
31–35	86 (24.0)	15 (35.7)	53 (24.3)	18 (18.4)		
36–40	39 (10.9)	5 (11.9)	22 (10.1)	12 (12.2)		
≥41	82 (22.9)	7 (16.7)	44 (20.2)	31 (31.6)		
Marital status					*χ*^2^ = 4.479	0.345
Married	255 (71.2)	27 (64.3)	155 (71.1)	73 (74.5)		
Unmarried	100 (27.9)	15 (35.7)	62 (28.4)	23 (23.5)		
Others	3 (0.8)	0 (0.0)	1 (0.5)	2 (2.0)		
Department					*χ*^2^ = 27.929	0.015
Internal medicine	63 (17.6)	11 (26.2)	37 (17.0)	15 (15.3)		
Surgery	46 (12.8)	5 (11.9)	25 (11.5)	16 (16.3)		
Emergency	29 (8.1)	6 (14.3)	22 (10.1)	1 (1.0)		
Pediatrics	33 (9.2)	4 (9.5)	22 (10.1)	7 (7.1)		
Gynecology	27 (7.5)	4 (9.5)	14 (6.4)	9 (9.2)		
ICU	48 (13.4)	3 (7.1)	37 (17.0)	8 (8.2)		
Operating room	14 (3.9)	2 (4.8)	9 (4.1)	3 (3.1)		
Other	98 (27.4)	7 (16.7)	52 (23.9)	39 (39.8)		
Educational level					*χ*^2^ = 2.650	0.851
High school/technical school	4 (1.1)	1 (2.4)	3 (1.4)	0 (0.0)		
Junior college degree	57 (15.9)	7 (16.7)	35 (16.1)	15 (15.3)		
Undergraduate degree	258 (72.1)	28 (66.7)	158 (72.5)	72 (73.5)		
Graduate degree and above	39 (10.9)	6 (14.3)	22 (10.1)	11 (11.2)		
Monthly income(RMB)					*χ*^2^ = 8.219	0.222
<4,000	38 (10.6)	2 (4.8)	28 (12.8)	8 (8.2)		
4,001–6,000	94 (26.3)	17 (40.5)	51 (23.4)	26 (26.5)		
6,001–8,000	94 (26.3)	11 (26.2)	59 (27.1)	24 (24.5)		
>8,000	132 (36.9)	12 (28.6)	80 (36.7)	40 (40.8)		
Work intensity					*χ*^2^ = 55.549	0.000
Very big	96 (26.8)	25 (59.5)	64 (29.4)	7 (7.1)		
Big	182 (50.8)	7 (16.7)	119 (54.6)	56 (57.1)		
Generally	79 (22.1)	10 (23.8)	34 (15.6)	35 (35.7)		
Small	1 (0.3)	0 (0.0)	1 (0.5)	0 (0.0)		
Family economy					*χ*^2^ = 28.382	0.000
Very nervous	33 (9.2)	10 (23.8)	21 (9.6)	2 (2.0)		
Nervous	98 (27.4)	14 (33.3)	67 (30.7)	17 (17.3)		
Generally	197 (55.0)	15 (35.7)	114 (52.3)	68 (69.4)		
Relatively wealthy	30 (8.4)	3 (7.1)	16 (7.3)	11 (11.2)		
Whether to engage in nursing again					*χ*^2^ = 42.629	0.000
Yes	71 (19.8)	6 (14.3)	33 (15.1)	32 (32.7)		
Consider	160 (44.7)	10 (23.8)	96 (44.0)	54 (55.1)		
No	127 (35.5)	26 (61.9)	89 (40.8)	12 (12.2)		
Psychological reward satisfaction scale score	3,83 (3.48,4.00)	3.71 (3.06,3.83)	3.75 (3.42,4.00)	4.00 (3.92,4.42)	H = 43.656	0.000

### 3.2 Results of latent profile analysis of subjective well-being among clinical nurses

In this study, latent profile analysis was conducted based on the 18 items of subjective well-being. Starting from the initial model, the number of classes was gradually increased, and latent profile models with 1 to 4 classes were sequentially fitted. The results showed that as the number of classes increased, the values of AIC, BIC, and aBIC continuously decreased, while the LMR for Model 4 was not statistically significant (*p* > 0.05). Based on a comprehensive comparison of the fit indices across all models, Model 3 was ultimately determined to be the optimal latent profile model, as shown in Table 2.

**Table 2:** Model fit indices for latent profile models of nurses’ subjective well-being.

Model	AIC	BIC	aBIC	Entropy	LMR (P)	BLRT (P)	Category probility
1	20997.529	21137.228	21023.019				
2	19815.595	20029.024	19854.538	0.883	0.0003	0.0000	0.634/0.366
3	19271.829	19558.988	19324.224	0.929	0.0203	0.0000	0.119/0.609/0.272
4	19082.316	19443.206	19148.165	0.947	0.3813	0.0000	0.087/0.604/0.035/0.274

Based on the scores of the three classes of clinical nurses’ subjective well-being across each item, the classes were named accordingly. Class 1 (C1) showed overall low scores; however, the scores for Item 3 (“Have you been able to maintain firm control over your behavior, thoughts, emotions, or feelings?”) and Item 13 (“Have you felt emotionally stable and in control of yourself?”) were significantly higher than those of other items. Since both Item 3 and Item 13 belong to the dimension of emotional and behavioral control, this class was named “Low Well-being-Emotional Control Group,” comprising 42 nurses (11.9%). Class 2 (C2) exhibited scores that fell between those of C1 and C3 across all items, with the prominent feature being the fluctuation in scores; therefore, it was named “Moderate Well-being-Overall Fluctuation Group,” comprising 218 nurses (61.0%). Class 3 (C3) showed overall scores at a good level; however, the score for Item 15 (“How concerned or worried are you about your health?”) was significantly lower than those of other items. Thus, this class was named “High Well-being-Health Concern Group,” comprising 98 nurses (27.2%). See Figure 1.

**Figure 1:**
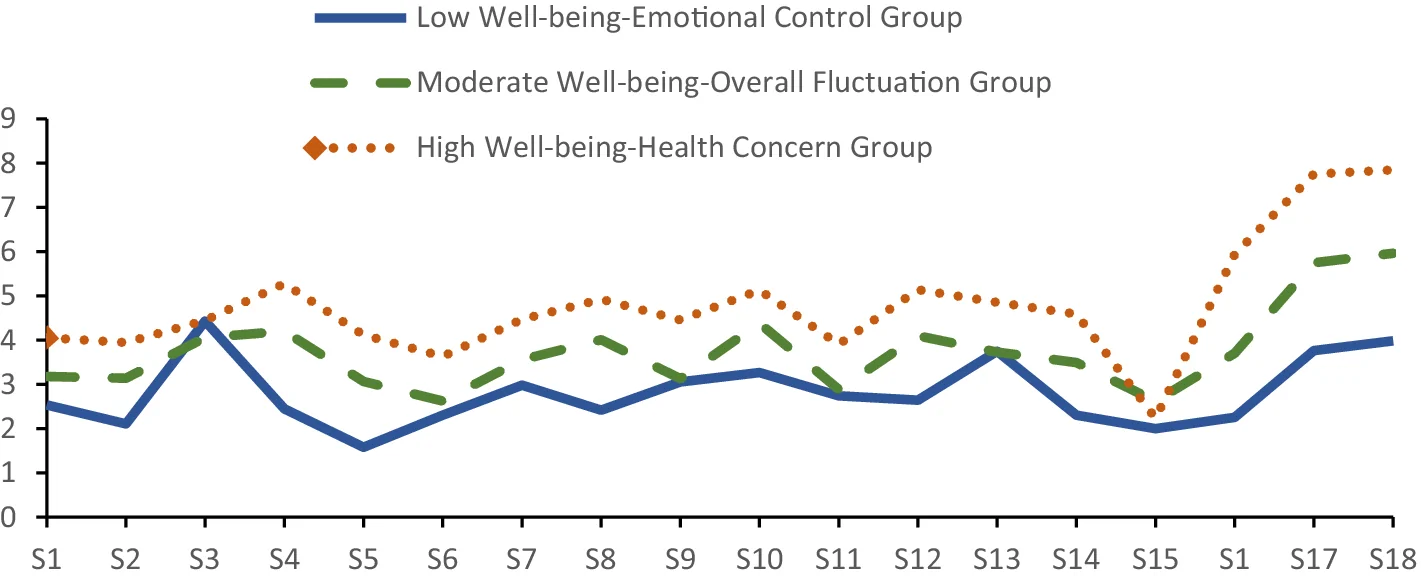
Latent profile characteristics of subjective well-being in clinical nurses.

### 3.3 Univariate analysis of latent classes of subjective well-being among clinical nurses

The results of this study showed that gender, department, work intensity, family economy, whether to engage in nursing again, and psychological reward satisfaction score differed significantly across the three classes of clinical nurses’ subjective well-being (*p* < 0.05), as shown in Table 1.

### 3.4 Multivariate analysis of latent classes of subjective well-being among clinical nurses

Using the High Well-being-Health Concern Group as the reference group and the other two groups as outcome variables, statistically significant variables from the univariate analysis were entered as independent variables into a multinomial logistic regression analysis. The assignment of independent variables is presented in Table 3. The results of logistic regression analysis showed that work intensity, family economy, department, whether to engage in nursing again, and psychological reward satisfaction were influencing factors of different latent profiles of clinical nurses’ subjective well-being, as shown in Table 4.

**Table 3:** The assignment of variables.

Variable	Assignment method
Gender	Male = 1; female = 2
Department	Intermal medicine = 1; surgery = 2; emergency = 3; pediatrics = 4;gynecology = 5; ICU = 6;operating room = 7; other = 8
Work intensity	Very big = 1; big = 2; generally = 3; small = 4
Family economy	Very nervous = 1; nervous = 2;generally = 3;relatively wealthy = 4
Whether to engage in nursing again	Yes = 1; consider = 2; no = 3
Psychological reward satisfaction scale score	The measured value

**Table 4:** Multivariate regression analysis of different class.

Items		*β*	SE	Wald *X*^2^	*P*	OR	95%CI
C1: C3	Intercept	37.635	2.896	168.873	0.000		
	Internal medicine	1.344	0.648	4.305	0.038	3.835	1.077 ~ 13.656
	Emergency	3.514	1.230	8.155	0.004	33.576	3.011 ~ 374.474
	Choose nursing as a career again	−1.801	0.644	7.821	0.005	0.165	0.047 ~ 0.583
	Consider choosing nursing as a career again	−2.381	0.538	19.595	0.000	0.092	0.032 ~ 0.265
	Family economy	−0.997	0.298	11.221	0.001	0.369	0.206 ~ 0.661
	Work intensity	−0.890	0.343	6.727	0.009	0.411	0.210 ~ 0.805
	Psychological reward satisfaction scale score	−0.143	0.038	14.440	0.000	0.867	0.805 ~ 0.933
C2: C3	Intercept	39.028	1.534	647.533	0.000		
	Emergency	2.549	1.085	5.516	0.019	12.799	1.525 ~ 107.424
	Choose nursing as a career again	−1.626	0.444	13.400	0.000	0.197	0.082 ~ 0.470
	Consider choosing nursing as a career again	−1.503	0.385	15.222	0.000	0.222	0.105 ~ 0.473
	Family economy	−0.538	0.213	6.347	0.012	0.584	0.384 ~ 0.887
	Work intensity	−0.571	0.231	6.104	0.013	0.565	0.359 ~ 0.889
	Psychological reward satisfaction scale score	−0.103	0.026	15.519	0.000	0.902	0.857 ~ 0.949

C1: low well-being-emotional control group. C2: moderate well-being-overall fluctuation group. C3: high well-being-health concern group.

## 4 Discussion

### 4.1 Heterogeneity of subjective well-being among clinical nurses

The results of this study showed that there was population heterogeneity in the level of nurses’ subjective well-being. ① Low Well-being-Emotional Control Group: Nurses in this group had relatively low overall scores on subjective well-being, but scored higher on the item “Have you been able to maintain firm control over your behavior, thoughts, emotions, or feelings?” This suggests that nurses in this group undertake a substantial amount of emotional control and regulation work in their daily practice. Prolonged engagement in inappropriate emotional labor may lead to job burnout, which affects the quality of clinical nursing and consequently reduces their level of well-being (20). ② Moderate Well-being-Overall Fluctuation Group: Nurses in this group were mainly characterized by dissatisfaction with life. This may be attributed to the heavy workload of nurses, which often makes it difficult to balance work and life. When time and energy cannot simultaneously meet the demands of both work and life, work–family conflict arises, leading to emotional exhaustion among nurses (21, 22). ③ High Well-being-Health Concern Group: Nurses in this group were mainly characterized by high energy levels, relative satisfaction with life, and the ability to maintain a pleasant mood at work. However, this group scored lower on the item “How concerned or worried are you about your health?” indicating that these nurses have a relatively high level of concern about their own health. This suggests that nursing managers should pay more attention to the Low Well-being with Emotional Control Group, adopt proactive and caring approaches, and provide targeted support and assistance to help them better manage negative emotions, gain positive experiences at work, and ultimately enhance their level of well-being.

### 4.2 Analysis of influencing factors of latent classes of subjective well-being among nurses

The results of this study found that, compared with nurses from other departments, emergency department nurses were more likely to be classified into the Low Well-being-Emotional Control Group and the Moderate Well-being-Overall Fluctuation Group, which is consistent with the results of Zhao (12). The reasons may be related to the fact that patients in the emergency department are critically ill, and emergency department nurses face heavy workloads, high work intensity, too little discretionary time at work, and an excessive number of non-nursing tasks (such as equipment maintenance and management, and high-standard demands from family members), all of which exacerbate the work pressure on emergency department nurses. At the same time, they also face interpersonal relationship pressures. As patients are in critical condition, family members are prone to anxiety, irritability, and other emotional reactions, often leading to communication barriers between nurses and family members (23). Hospital administrators may consider increasing staffing levels in the emergency department, optimizing shift scheduling and workload distribution, and minimizing overtime hours, all of which could help reduce the work pressure experienced by emergency nurses.

This study showed that nurses who would choose or consider choosing nursing as a career again were more likely to be classified into the High Well-being-Health Concern Group. A plausible explanation is that nurses who express a willingness to choose nursing as a career again tend to possess clearer career goals and a more explicit development trajectory. Such clarity may drive them to engage in ongoing learning and self-improvement, which in turn strengthens their sense of professional identity within the nursing field. Previous studies (24) have shown that a strong sense of professional identity is associated with greater work enthusiasm and initiative, improved job performance and career satisfaction, and enhanced career stability and professional growth. Nursing administrators consider helping nurses clarify their career goals and strengthen their sense of professional identity and belonging by providing career development opportunities and strengthening career planning guidance.

This study showed that nurses with a comfortable family financial status were more likely to be classified into the High Well-being-Health Concern Group, which is consistent with the findings of Sun (25). Based on social exchange theory, one possible explanation is that a good family financial status, as a hygiene factor, provides nurses with greater material enjoyment and enables them to perceive more positive emotions, thus leading to higher levels of well-being (26). Hospital administrators should pay more attention to nurses with strained family financial situations and improve the salary system for nursing staff. The top-level design to strengthen the embodiment of the technical and labor value of nursing staff may serve as a potential intervention target. It is recommended that different salary grades be established based on factors such as the degree of risk, technical complexity, and workload associated with various nursing positions, so that nurses’ income levels are aligned with their technical proficiency and work burden.

This study found that nurses with high work intensity were more likely to be classified into the Low Well-being-Emotional Control Group, which is similar to the findings of Peršolja (27). With the promotion of high-quality nursing services, higher demands have been placed on clinical nursing work. Nurses are not merely passive executors of medical orders but also assume multiple roles such as educators, consultants, and researchers. Coupled with the continuous increase in hospital bed capacity and other factors, nursing workloads have greatly increased, thereby reducing nurses’ subjective well-being. This suggests that relevant hospital departments may consider increasing the number of nursing staff, rationally allocate nursing human resources, further meet the national requirements for the bed-to-nurse ratio, and alleviate nurses’ workload.

This study found that nurses with higher psychological reward satisfaction were more likely to be classified into the High Well-being-Health Concern Group, which is consistent with the results of Ren (28). Psychological reward satisfaction is a subjective emotional experience at the individual level, and psychological rewards from doctors and head nurses can influence nurses’ work attitudes and behaviors (29). Previous studies have shown (8, 30) that nurses’ psychological reward satisfaction is positively correlated with job performance, work enthusiasm, work engagement, and work attitudes. Encouragement and recognition from managers are key to stimulating nurses’ work initiative (31). When nurses receive praise and encouragement from head nurses, doctors, colleagues, and patients, their higher-level needs—such as belonging and love, respect, and self-actualization—are satisfied, thereby enhancing their well-being (32). Nursing managers should recognize the importance of psychological rewards in improving nurses’ subjective well-being and provide psychological rewards to nurses at appropriate times. At the same time, managers should also encourage nurses’ collaborators, such as doctors, technicians, and family members, to actively express approval and gratitude to nurses, thereby enhancing nurses’ psychological rewards.

## 5 Conclusion

This study found that nurses’ subjective well-being could be classified into three latent profiles: the Low Well-being-Emotional Control Group, the Moderate Well-being-Overall Fluctuation Group, and the High Well-being-Health Concern Group. Department, willingness to choose nursing again, family finanical status, work intensity, and psychological reward satisfaction were variables significantly associated with latent profile membership of subjective well-being among nurses. The findings of this study provide a hypothetical foundation for further longitudinal tracking and intervention research; however, the causal direction and intervention effects need to be verified through prospective designs.

### 5.1 Limitation

This study was cross-sectional in design; therefore, causal relationships among variables cannot be inferred. The survey participants were nurses from tertiary hospitals in Weifang, Shandong Province, and nurses working in primary-level hospitals or long-term care facilities were not included. In addition, all data were collected via self-administered questionnaires, which may be susceptible to respondent bias. Future research could expand the scope of investigation to include a broader range of hospital types and institutions, and adopt mixed-method designs to further explore the mechanisms through which additional influencing factors affect subjective well-being.

## Data Availability

The original contributions presented in the study are included in the article/supplementary material, further inquiries can be directed to the corresponding author.
